# Modulatory activity of testosterone on growth pattern and IGF-1 levels in vanishing testis syndrome: a case report during 15 years of follow-up

**DOI:** 10.1186/s12902-022-01258-2

**Published:** 2023-01-12

**Authors:** Marta Franco, Keyvan Khorrami Chokami, Manuela Albertelli, Claudia Teti, Francesco Cocchiara, Federico Gatto, Carlo Trombetta, Diego Ferone, Mara Boschetti

**Affiliations:** 1 Endocrinology Unit, UOSD Azienda Ospedaliera San Camillo-Forlanini, Rome, Italy; 2 Endocrinology Unit, Department of Internal Medicine & Medical Specialties (DiMI), IRCCS Ospedale Policlinico San Martino, University of Genoa, Viale Benedetto XV, 16132 Genoa, Italy; 3 Endocrinology, Diabetology and Metabolic Diseases Unit, ASL1 Imperia, Italy; 4 Endocrinology Unit, IRCCS Ospedale Policlinico San Martino, Genoa, Italy; 5 Department of Medicine, Surgery and Health Sciences, Urological Clinic, University of Trieste, Trieste, Italy

**Keywords:** Growth, Puberty, Hypogonadism, IGF-1, Testosterone, Case report

## Abstract

**Background:**

The vanishing testis syndrome (VTS), is a 46, XY disorder of sex development (46, XY DSD) and is characterized by the absence of testis in a 46, XY subject with male genitalia, gonadal dysgenesis and consequent hypergonadotropic hypogonadism.

**Case presentation:**

A young man affected by VTS has been followed up for more than 15-year in our center. The patient received different testosterone formulations, which modulated his IGF-1 levels and height velocity, depending on different stimulatory effects, mimicking pubertal spurt until achieving a final height in line with his genetic target.

Exogenous testosterone, activating GH/IGF-1 system, can directly influence growth pattern. With this particular case report we demonstrate that an accurate monitoring of patients with VTS, as well as a perfect reproduction of testosterone secretion during pubertal spurt, can guarantee a normal growth and development and, consequently, a high level of quality of life in adulthood.

**Conclusion:**

Testosterone levels act an important role during pubertal spurt in modulating the GH/IGF-1 axis, besides its well-known impact in sexual development.

Very little amount of exogenous testosterone can stimulate IGF-1 secretion and provide to growth velocity the drive that characterizes the initial phases of the growth spurt.

## Background

The vanishing testis syndrome (VTS), also known as testicular regression syndrome, is characterized by the absence of one or both testicles in a 46, XY individual and, as a cause of non-palpable testes, it is classified among disorders of sex development (46, XY DSD) [1].

VTS is one of the most common causes (35–60%) of non-palpable testes in children [2–5], probably caused by the atrophy and disappearance in fetal life of an initially normal testis. The phenotype of these patients is characterized by the presence of external male genitalia and Wolffian duct with unilateral or bilateral impalpable testes. These features indicate a possible damage of the testes occurred in utero, or perinatally, after a normal transabdominal testicular migration, associated with a functioning testicular tissue during the first 8–16 weeks of pregnancy [6–10]. The etiology is largely discussed [11, 12], however VTS seems caused by the loss of blood supply to the testicle (vascular thrombosis, torsion) as a perinatal event [13]. A vascular accident or a prenatal torsion rather than an endocrinopathy are possible explanation of the physiopathology of a fibrotic nodule that is commonly observed at the edge of the spermatic cord. This peculiar finding of the VTS is associated also with dystrophic calcifications and hemosiderin deposition [14]. The direct endocrine consequence of the gonadal dysgenesis is hypergonadotropic hypogonadism, and the lack of testosterone negatively affect the normal pubertal spurt. These patients, in adulthood, may display a normal or increased height without pubertal growth spurt [15–17]. Sex hormones (SHs) impact growth spurt via their effect on GH/IGF-1 axis. Briefly, the initial small increase of SHs stimulates GH secretion in the early phase of puberty, whereas during late puberty, the further increase of SHs determines bone ossification and, therefore, the arrest of growth [18]. In order to mimic the natural increase of SHs and their effect on GH/IGF-1 axis and skeletal growth, male patients with DSD and hypogonadism need to start testosterone therapy during pubertal age. The hormone replacement therapy deserves particular attention due to the specific activities of testosterone in this critical phase. We report the effects of different testosterone formulations on growth and pubertal development in a young man with VTS followed in our Endocrinology Unit over a period of more than 15 years.

## Case presentation

A 28-month-old child affected by VTS was referred to our Medical Center for endocrine evaluation, treatment and follow-up.The patient was born at 40^th^ week of gestation (birth weight 3.695 kg, length 54 cm, head circumference 35 cm). He had normal-appearing external male genitalia except the small testes and a short frenulum. He was breastfed until 3 months of age. No significant medical events were reported during infancy. The testes were palpable in the scrotum until the age of fifth months, and they were impalpable when the child turned one year, as reported in the pediatrician’s medical records and in the first urological evaluation performed in another Medical Center.

The karyotype was male (46 XY) and the ultrasonography confirmed the absence of testes in the scrotum, suggesting a potential case of cryptorchidism. Therefore, at the age of two, the patient underwent a surgical inguinal exploration and a blind ending of spermatic vessels and vas deferens were found in the right inguinal canal. In the left one a gubernaculum was identified, vessels and vas deferens ended in a fibrotic remnant, which was removed. A frenulectomy was also performed and bilateral testicular prostheses were placed. A topic formulation of dihydrotestosterone (DHT) was started in order to improve scrotal trophism and capacity.

### Physical examination

**Fig. 1:**
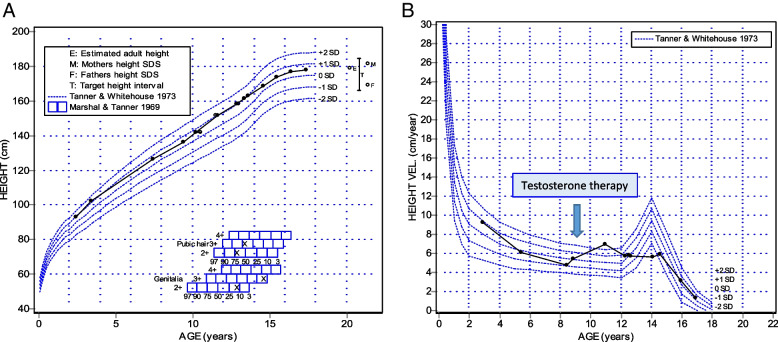
Growth pattern of the patient. Legend: Height (panel **A**) and height velocity (panel **B**) are plotted against the normal population standards, according to Tanner

At the first evaluation in our Endocrinology Unit the child was 28 months old. He was 93 cm tall (0.7 SDS) and he weighed 14.5 kg (0.9 SDS). Clinical examination and hormonal evaluation did not show any pathological sign. The data of regular yearly follow-up, including auxological and hormonal data, were recorded (Table 1 and Fig. 1).

**Table 1:** Patient’s relevant clinical data during the 15 years of follow-up

Date	Age yrs	Bone Age	Height cm	Height SDS	Height velocity SDS	Weight SDS	IGF-I ng/ml	IGF SDS	T ng/ml	Pubertal stage	Therapy
28/06/91	2.4	-	93	0.78	-	0.2	36	-0.75	< 0.2	Ph0 G0	-
19/06/92	3.38	-	102	0.98	0.9	0.6	50	-0.53	< 0.2	-	-
03/07/96	7.42	-	126.6	0.57	-0.2	-0.2	49	-0.96	< 0.2	Ph1 G1	-
19/06/98	9.38	-	136	0.28	-0.9	0.4	65	-0.81	< 0.2	-	TPr 2.5 mg die
23/04/99	10.22	10.5	141.8	0.80	2.4	0.4	151	0.02	0.8	-	TPr 5 mg die
24/09/00	11.64	11.6	151.7	1.01	2.8	0.2	336	0.47	0.2	-	TUn 20 mg die
06/12/01	12.84	13	158.5	0.65	0.4	2.0	236	-0.14	0.37	Ph2 G2	TUn 40 mg die
11/06/02	13.36	13.5	161.6	0.58	-0.7	0.9	287	0.17	0.37	Ph3 G2	TUn 40 mg die
05/09/03	14.59	14.7	168.4	0.53	-2.9	1.1	375	0.71	0.6	Ph3 G3	TUn 40 mg die
16/07/04	15.45	15.6	173.7	0.46	0.3	1.2	379	0.74	2.2	-	TE ½ fl 21 days
14/06/05	16.36	16.5	176.6	0.46	0.3	1.2	352	0.57	2.96	Ph4 G4	TE 1 fl 21 days
12/06/06	17.36	17.5	177.9	0.51	0.1	1.4	405	0.9	7.6	Ph5 G5	T Gel daily

*Legend yrs* years, bone age (Greulich-Pyle calculated), *SDS* standard deviation score, *T *serum total testosterone, *TPr* testosterone propionate, *TUn *testosterone undecanoate, *TE *testosterone esters, *T Gel* testosterone gel

### Laboratory Examination

At 4 years, LH and FSH were undetectable (both 0.00 mUI/L) and the GH response to GHRH + Arginine test was normal.

At the age of 9.38 years (Table 1), an increase of LH levels (3.38 mUI/L) and a simultaneous decrease of height velocity (-0.9 SDS, height 136 cm) were recorded.

### Final diagnosis

Growth and puberty delay in a patient affected by VTS-related anorchia.

### Treatment

At the age of 10, oral treatment with testosterone propionate (2.5 mg/day) was started and carried on for 10 months; the dose was subsequently increased to 5 mg/day. Height and growth velocity increased proportionally with serum testosterone and IGF-1 levels (Fig. 1). At the age of 11.64 the growth velocity was above normal values for age (SDS 2.8). Therefore, testosterone propionate was withdrawn and the less active testosterone undecanoate formulation (20 mg/die) was initiated, aiming to reduce the androgen effect on bone maturation. Consequently, growth velocity decreased in the following years (SDS 0.4) and the oral dose of testosterone undecanoate was increased up to 40 mg. At the age of 15.4 testosterone treatment was switched to the intramuscular formulation (Table 1). Growth velocity was 6 cm/year until the age of 15, and then it dropped to 3.2 cm/year. No other therapies were administered.

### Outcome and follow up

Anthropometric and clinical parameters, biochemical data and bone age were regularly assessed during the follow-up. Bone age remained always comparable with chronological age. The final height was 177.9 cm in line with the parental target (169.8 cm—180.6 cm). Androgenization was completed when the patient turned 15. During the follow-up, testosterone levels changed according to the different modalities of administration (Table 1), whereas IGF-1 remained within the normal range for age. The growth pattern ran parallel to the IGF-1 concentration and the patient's stature reached the highest percentile according to his parental target.

## Discussion

The VTS, or testicular regression syndrome, is the most common cause of non-palpable testes among cryptorchidism cases [5]. To date, because of the lack of clear consensus and/or evidence-based guidelines, there is a high degree of variability in the diagnostic approach as well as clinical management of these patients [19]. The removal of the fibrotic remnant/nodule is controversial and the discussion concerns the rare possibility to find germinal cells in the remnant tissue that represents a risk of cancer development [2, 14, 20, 21].

In case of non-palpable testes, it is also debated whether the inguinal exploration and the laparoscopic abdominal exploration should both be performed in all cases [3, 22]. The European Association of Urology/European Society for Paediatric Urology guidelines suggest that inguinal exploration may be avoided if blind ending vessels are observed during diagnostic laparoscopy. Vice versa, laparoscopy may be omitted if during inguinal exploration a nubbin is found and removed [23].

In our case the patient was firstly evaluated by an ultrasound, which demonstrated the absence of testicles in the scrotum. Considering that ultrasonography could be misleading [24], the patient underwent inguino-scrotal exploration and a fibrotic remnant was removed.

VTS lead to a condition of anorchia with a related hypergonadotropic hypogonadism. In this context replacement therapy with testosterone should be started at pubertal age, normally between 11 and 14 years [25]. To date, there are different preparations of testosterone: oral testosterone (not available in USA, because of liver toxicity), buccal administration with mucoadhesive tablets applied to the gums, nasal gel formulation, subdermal testosterone pellets. The transdermal patches or gel are easy to use, although requiring a daily administration, are generally well accepted by the patients and allow mimicking the physiological circadian rhythm of testosterone. Finally, intramuscular formulation of testosterone esters (testosterone cypionate-TC, enanthate-TE, undecanoate-TU), which requires less frequent administrations than topical, have the disadvantage of the intramuscular (IM) route and, thus, the risk of inflammation and/or pain in the injection site [26, 27].

In the present case we started testosterone replacement therapy once the decrease of growth velocity was running in parallel with a significant increase of circulating LH levels. Once GH deficiency was ruled out, a low dose of oral testosterone was started in order to mimic the initial response of the Leydig cells to the pituitary stimulus. Later, the oral one was replaced by the intramuscular formulation, which was also preferred by the patient.

The relationship between SHs and the GH/IGF-1 axis is well known since many years. However, the precise mechanism of action of SHs in the induction of statural growth spurt is still debated. It has been demonstrated that androgens can stimulate the secretion of GH and the subsequent increase of IGF-1 levels [28]. Many studies have postulated that testosterone exerts its growth-promoting effect by enhancing the secretory pattern of GH [29], which in turns stimulates longitudinal bone growth either indirectly, via hepatic IGF-1 secretion [30], or directly at the site of the epiphyseal growth plate, probably through the local production of IGF-1 [31]. Moreover, an indirect effect of testosterone through the activation of GH/IGF-1 has been shown by the evidence that pretreatment with testosterone (or estradiol in female) increases GH response to stimuli. This indirect effect of testosterone is supported by the observation that this sex hormone exerts no significant increase in body length in the absence of GH (e.g. in hypophysectomized rats) [32, 33].

However, androgens can also directly stimulate the skeletal growth in patients affected by GH deficiency [34]. In particular, androgens have direct effect on the growth plate in stimulating longitudinal growth, and estrogens (derived from androgen aromatization) lead to epiphysis fusion [35]. Moreover, Yoshizawa and colleagues have also found that the combination of testosterone and IGF-1 can modulate the synthesis and secretion of some IGFBPs [36].

To date, no data are available about the growth pattern in subjects followed longitudinally since infancy until final height has been reached.

We noticed that the dose of testosterone as small as 0.08 mg/kg/day, which produces a small increase of the circulating hormone, could stimulate IGF-1 to reach pubertal concentrations and increase growth velocity to values comparable to the peak growth velocity (Table 1). Since this phenomenon occurred in our patient earlier than expected, we modified the treatment by replacing testosterone propionate with the less active undecanoate. This switch produced a flattening of the growth curve and prevented a premature ossification of the growth plate allowing a normal final height.

## Conclusion

A very small amount of testosterone can stimulate IGF-1 secretion and enhance growth velocity, which characterizes the initial phases of the growth spurt. When the diagnosis of hypogonadism is certainly made, as in the present case, testosterone therapy could be probably earlier started than suggested in the guidelines and gradually titrated. Patients should be carefully monitored and any physical changes be recorded. The aim is to guarantee to these patients a normal growth and sexual development, namely an adequate standard of quality of life in adulthood.

## Data Availability

All the data generated and/or analyzed during this study are included in this published article.

## Abbreviations

VTS: Vanishing testis syndrome
SHs: Sex hormones
DHT: Dihydrotestosterone
SDS: Standard deviation score
IGF-1: Insulin-like growth factor-1
GH: Growth-hormone
IGFBPs: Insulin-like growth factor-binding proteins
